# The Neutralizing Antibody Responses of Individuals That Spontaneously Resolve Hepatitis C Virus Infection

**DOI:** 10.3390/v14071391

**Published:** 2022-06-25

**Authors:** Vanessa M. Cowton, James I. Dunlop, Sarah J. Cole, Rachael E. Swann, Arvind H. Patel

**Affiliations:** 1 MRC-University of Glasgow Centre for Virus Research, University of Glasgow, Glasgow G61 1QH, UK; james.dunlop@glasgow.ac.uk (J.I.D.); sarah.cole@glasgow.ac.uk (S.J.C.); rachael.swann@ggc.scot.nhs.uk (R.E.S.); arvind.patel@glasgow.ac.uk (A.H.P.); 2 Department of Gastroenterology, Queen Elizabeth University Hospital, Glasgow G51 4TF, UK

**Keywords:** hepatitis C, spontaneous clearance, neutralizing antibody

## Abstract

Hepatitis C virus (HCV) infection is a major global health problem. In the majority of cases the virus is not cleared by the host immune response and progresses to chronic infection. Studies of the neutralizing antibody responses in individuals that naturally clear infection are limited. Understanding what constitutes a successful antibody response versus one that has ‘failed’ and resulted in chronic infection is important to understand what type of antibody response would need to be elicited by a protective vaccine. Samples from spontaneous clearers are difficult to obtain therefore studies are often limited. In our study through HCV Research UK, we had access to a cohort of over 200 samples. We identified the samples that contained HCV neutralizing antibodies using ELISA and HCV pseudoparticle (HCVpp) assays. We then utilised mutagenesis and cross-competition analysis to determine the profile of the neutralizing antibody responses. In addition, we analysed a cohort of samples from chronic infection using the same techniques to enable direct comparison of the antibody profiles observed in both cohorts. We conclude that similar profiles are present in both cohorts indicating that it is not the neutralizing antibody response per se that determines the outcome of infection. These data will provide useful information for future HCV vaccine design.

## 1. Introduction

Hepatitis C virus (HCV) is a positive-sense RNA virus within the Flaviviridae family. The virus is extremely diverse and is classified into seven distinct genotypes and 67 subtypes [1]. Globally an estimated 70 million people are infected with HCV contributing to around 400,000 deaths annually. In recent times significant advances have been made in the development of very effective direct-acting antiviral drugs (DAAs) that can clear viral infection. However, due to the silent nature of the initial infection which is often asymptomatic many of those infected remain undiagnosed. Additionally, a significant proportion of infected individuals live in the developing world and do not have access to DAA treatment. Therefore, development of a protective vaccine is important to contribute to the prevention and eradication of this disease.

In natural infections, roughly 20–25% clear acute infection and 75–80% proceed to chronic infection [2,3]. Factors including female gender, younger age of infection, co-infection with hepatitis B virus (HBV) and specific alleles of the Il-28B (interferon-λ3) and HLA class II genes are associated with spontaneous resolution of infection [4,5,6]. While T-cell responses have long been demonstrated to be associated with spontaneous clearance [2,7,8], the role of neutralizing antibody (nAb) responses is less clear. An early study failed to detect nAbs in five out of seven individuals that spontaneously cleared the virus [9]. More recently there is evidence that an early neutralizing antibody response is involved in spontaneous clearance [10,11]. Classical spontaneous clearance refers to clearance during the acute phase of infection (6–24 months) post-infection, however spontaneous clearance of chronic infection is often overlooked despite multiple reports in the literature [12,13,14,15,16,17,18]. In many cases, clearance of chronic infection has been associated with another event including infection with other hepatitis viruses, surgery or withdrawal of antiretroviral therapy (ART). Bulteel et al. (2016) concluded that similar to spontaneous clearance during acute infection, clearance of chronic infection was also positively associated with female gender, younger age of infection and co-infection with HBV [17].

In order to inform vaccine design, it is important to understand nAb responses during spontaneous clearance. In this study we have investigated the antibody responses in a cohort of individuals that have cleared HCV infection without treatment. We compared these responses to those from individuals that have chronic HCV infection to understand if the antibody profiles are fundamentally different between these two groups. We show that the neutralizing antibodies produced by both groups target the same domains of the HCV E2 glycoprotein. Our data indicate that it is not the nature of the antibody response per se that determines outcome of infection.

## 2. Materials and Methods

### 2.1. Patient Characteristics of Spontaneous Resolver Cohort

Serum samples for the study were requested from the HCV Research UK Clinical Database and Biobank [19]. The cohort has >10,000 individuals recruited from 56 centres across the UK. The biobank provided us with 233 serum samples from individuals that had been identified in medical records as having cleared HCV infection without treatment. Ethical approval for the study was granted by the HCV Research UK Tissue and Data Access Committee.

### 2.2. Patient Characteristics of Chronic HCV (CHCV) Cohort

Subjects infected with chronic genotype (gt) 1 or gt 3 HCV were recruited from three local liver clinics. Those individuals with BMI over 30, co-existing liver pathologies or hepatocellular carcinoma were excluded from the study. Also recruited were healthy individuals with no liver pathologies or significant co-morbidities. All subjects completed a symptom questionnaire, baseline biochemistry, IL-28B profiles and virology, and clinical details were checked and recorded. Serum and whole blood samples were obtained and stored at −70 °C. Ethical approval was granted for this study by regional ethics committees and all patients gave informed consent.

### 2.3. Cell Lines

Human hepatoma Huh-7 cells and HEK-293T cells were grown in Dulbecco’s modified Eagle’s medium supplemented with 10% foetal calf serum, 5% nonessential amino acids and 200 mM l-glutamine.

### 2.4. Antibodies

The HCV anti-E2 human monoclonal antibodies (HmAb) CBH-4B, CBH-7, HC-1, HC-11 and HC33.1 have been described previously [20,21] and were a generous gift from Steven Foung. The sequences of the heavy and light chain sequences of monoclonal antibody 1:7 were obtained from a publish patent, synthesized and subcloned into the appropriate pFuse-ss human IgG vectors (Invivogen, San Diego, USA) [22]. The plasmids were verified by Sanger sequencing then expressed in the Expi293 expression system as per the manufacturers’ instructions (ThermoFisher Scientific, Waltham, MA, USA). After 3–5 days the medium was harvested, and IgG was purified using a HiTrap Protein G column on an ÄKTA Pure system (Cytiva, Marlborough, MA, USA).

### 2.5. IgG Purification

Triton X-100 was added to patient serum samples at a final concentration of 0.05% (*v*/*v*) and IgGs therein purified using Protein G IgG purification spin columns (ThermoFisher Scientific, Waltham, MA, USA). The final concentration was determined using a Nanodrop 1000 spectrophotometer (ThermoFisher Scientific, Waltham, MA, USA).

### 2.6. GNA Capture Assay

The ELISA to detect antibody binding to E1 and E2 glycoproteins was performed as described previously [23]. HEK-293T cells were transfected with plasmid expressing H77 Gt1a E1E2, then incubated for three days. The E1E2 glycoproteins present in the cell lysates were captured on GNA (Galanthus nivalis agglutinin) lectin coated Immulon 2HB enzyme immunoassay plates (ThermoFisher Scientific, Waltham, MA, USA). Patient sera were added at 1:500 dilution in PBS-T and bound antibodies were detected using HRP-conjugated anti-human IgG antibody (A0170, Sigma-Aldrich, St. Louis, MO, USA) and TMB (3,3′, 5,5′-tetramethylbenzidine, Thermofisher Scientific, Waltham, MA, USA) substrate. A positive control serum from an individual with chronic HCV infection was included on all plates. Absorbance values were measured at 450 nm and normalized according to the positive control, to enable comparison of separate ELISA plates. Binding to HEK-293T lysate lacking HCV glycoproteins was used as a control to eliminate false positives.

### 2.7. Generation of HCV Pseudoparticles (HCVpp) and Neutralization Assays

The Gt1 E1E2 HCVpp panel is composed of 11 diverse Gt1 HCVpp, as we described previously [24]. HEK-293T cells were co-transfected with plasmids expressing MLV Gag-pol, the MLV transfer vector carrying firefly luciferase reporter and HCV E1E2. After 72 h, the medium was harvested, filtered through a 0.45 µM membrane and used as a source of HCVpp as described previously [25]. For neutralization assays HCVpp and purified human IgG at 100 µg/mL were incubated together for 1 h at 37 °C, and then the mixture used to infect Huh7 cells for 3 h. The HCVpp-IgG mix was removed, and fresh media was added. At 3 days post-infection, cells were lysed and luciferase activity measured using the GloLysis Luciferase substrate assay (Promega, Madison, WI, USA).

### 2.8. Mutagenesis of E2 Epitopes

E1E2 mutants L413A, W420A, W529A and G530A have been described previously [26]. Site-directed mutagenesis PCR using the QuikChange II kit (Agilent, Santa Clara, CA, USA) was used to generate H77 gt1a E1E2 containing alanine substitutions at positions E2 L441 and F442. The sequence of E1E2 L441A and E1E2 L442A clones was verified by sanger sequencing. The panel of mutant E1E2 cell lysates was produced by transfecting 8µg of each plasmid into HEK-293T cells. The cells were lysed after 72 h.

### 2.9. Normalisation ELISA for Mutant Lysates

Lysate was added to Immulon 2HB plates (Thermo Scientific) coated with GNA and incubated at RT for 2 h. Anti-E2 mouse monoclonal (mAb) antibody ALP98 at 0.1 µg/mL was added and incubated for 1 h. Bound ALP98 was detected with 1:1000 anti-mouse-HRP (A4416, Sigma-Aldrich, St. Louis, MO, USA) then TMB reagent. Plates were washed in PBS-T (x3) between steps. Absorbance was measured at A450 nm.

### 2.10. Mutagenesis ELISA Assay

The GNA ELISA was adapted to detect binding to the E1E2 mutant panel. The normalised lysate was added to Immulon 2HB plates coated with GNA and incubated for 2 h. For each sample 20 µg/mL purified IgG was added and incubated for 1 h to allow binding to E1E2. A positive control of 0.1 µg/mL mouse mAb ALP98 was also included. The plates were washed in PBS-T (x3) then secondary anti-human-HRP antibody (A0170 Sigma, 1:5000) or anti-mouse-HRP (A4416 Sigma, 1:1000) was added for 1 hr. The plates were washed again in PBS-T (x6) then developed with TMB substrate. Absorbance was read at 450 nm.

### 2.11. Epitope Targeting by Cross-Competition Assay

Soluble gt 1a E2 (H77) protein (sE2) was purified following expression in High Five insect cells. Briefly, cells were infected with recombinant baculovirus expressing sE2 and harvested after 4 days. sE2 protein was purified from the supernatant via a His-tag using affinity chromatography on a Ni-NTA column. The sE2 at 1 µg/mL was used to coat Immulon 2HB plates then incubated with purified patient IgG at 200 µg/mL in PBS-T. The biotinylated antibodies to known epitopes were then added at a concentration close to their EC50 [27,28,29,30]. Finally, Streptavidin-HRP was added, binding was detected using TMB substrate by measuring the absorbance at 450 nm. The reduction in the relative binding of each biotinylated antibody (calculated as percentage reduction in absorbance) on addition of patient IgG compared to the PBS-T control was determined.

### 2.12. Analysis

The parametric t-tests and correlation statistical analysis was performed using GraphPad Prism 6 Software (GraphPad Software, San Diego, CA, USA) and SPSS v. 19.09 (IBM, New York, NY, USA).

## 3. Results

A retrospective cohort (SR cohort) of 233 samples from HCV spontaneous resolvers collected from 28 centres across the UK was provided by HCV Research UK [19]. All individuals included in the study have been clinically diagnosed as spontaneous clearers and critically, have never received any treatment for HCV. The demographics for the cohort are shown in Table 1. As expected for the UK, the cohort is predominantly Caucasian (90.6%) and two-thirds of the cohort are male (66.7%). The main route of infection is through intravenous drug use (67.9%). The mean age at sample collection is 45.6 yrs (range 20–80 yrs). Due to the retrospective nature of the study the clinical data for all subjects is incomplete, where it is known, the median duration from the first positive HCV test to serum collection is 5.5 yrs (range 0–23.1 yrs, no data for 13 samples) and the median duration from the first negative HCV test to serum collection is 0.38 yrs (range 0–19.9 yrs, no data for 114 samples). The median duration from confirmed positive HCV test to negative HCV test is 2.1 yrs (range 0–20.2 yrs, no data for 119 samples). Unfortunately, in many cases the true date of infection and indeed clearance could not be estimated, as a result it is difficult to determine if individuals cleared infection during the acute or chronic phase. Therefore, we did not attempt to separate the samples further into these groups The majority of samples were not genotyped, however, for the small group that we do have data for, as expected for the UK, they are evenly distributed between Gt1 and Gt3, There was also one Gt2 and one Gt4 sample.

### 3.1. E1E2 Binding of Spontaneous Resolver Cohort

**Figure 1:**
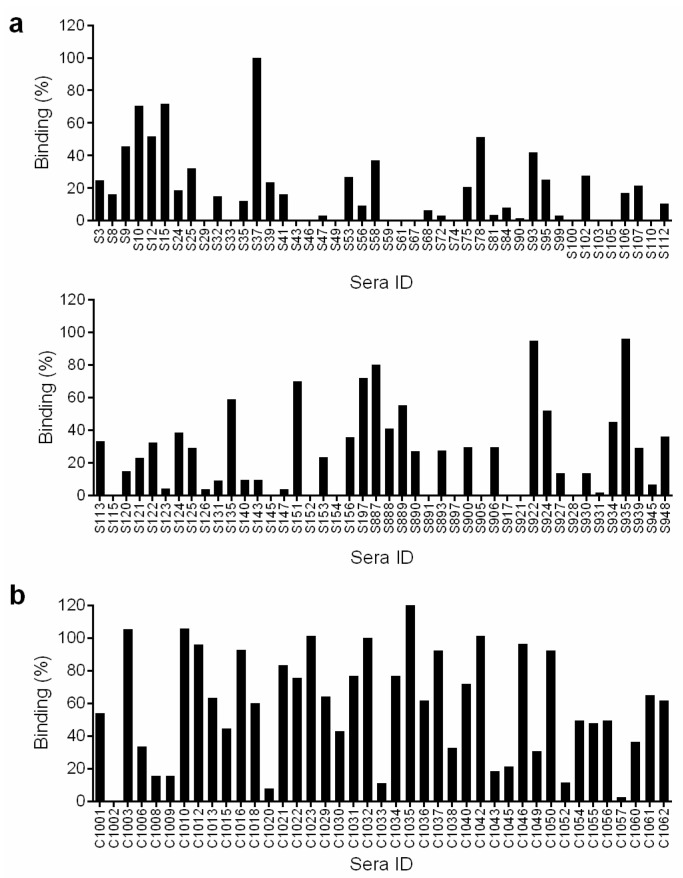
*Binding to E1E2 lysate.* Serum samples diluted 1:500 were screened for binding to gt1a H77 E1E2 lysate in a GNA ELISA assay. The binding activity is expressed as a percentage relative to binding of a control HCV sera sample and is adjusted for binding to control lysate with no E1E2. Values shown are the mean of two independent replicate experiments. (**a**) Sera from the spontaneous resolver cohort. (**b**) Sera from the chronic HCV cohort.

In order to identify samples that had detectable levels of HCV antibodies we screened the sera for HCV E1E2 binding antibodies by ELISA. To setup the assay we compared two dilutions of sera (1:500 and 1:1000) and different concentrations of secondary antibody. We found that 1:500 sera dilution and 1:10,000 secondary antibody were optimal. A 1:500 sera dilution is roughly equivalent to 15–44 ng IgG based on the average range of IgG present in human sera (7.5–22 mg/mL). The binding to gt1a E1E2 strain H77 lysate was assessed for all the samples. The HCV genotype was not known for the majority of the samples therefore we chose to use strain H77 E1E2 for the screen. It is possible that we may have missed some positive sera in the screen although in our experience E1E2 antibodies present in sera from gt1–6 infected individuals can bind to strain H77 E1E2. Indeed, our positive sera group includes samples from individuals infected with HCV gt 1,2,3 and 4. A positive control sera from a chronic HCV infection was included on all ELISA plates, to enable comparison between experiments. Eighty-eight samples from the cohort tested positive for binding to H77 E1E2, these were subsequently screened for false positives by testing binding to negative control lysate that does not contain HCV E1E2. Forty-nine samples (21%) had >10% E1E2 binding relative to the positive control, thereby confirming the presence of HCV E1E2 antibodies in these samples (Figure 1a). The clinical data of these samples is provided in Supplementary Materials Table S1.

### 3.2. Neutralization Profile of Spontaneous Resolver Cohort

To investigate the neutralizing capacity of the spontaneous clearer samples we monitored neutralization of a panel of eleven representative gt1 panel of HCVpp which we had reported previously [24]. Neutralization was assessed using purified IgG to avoid the possibility of anomalies caused by other sera components. This also allowed a standard amount of IgG to be tested although the actual levels of E1E2 binding antibodies within the IgG population was not measured. In total, about 50% (24/49) of samples could neutralize at least one HCVpp isolate by at least 50% (Table 2). However, only five serum samples were classified as broadly-neutralizing i.e., able to neutralize six or more members of the panel by at least 50%.

### 3.3. Comparison with Sera from HCV Chronic Infection

We were interested to determine how the results obtained from the spontaneous resolver cohort would compare in the same assays to sera from individuals with chronic HCV infection. To address this question, we selected a small cohort of 41 serum samples from the Glasgow chronic HCV cohort [24]. This had similar demographics to the spontaneous clearer cohort. The cohort was predominantly male Caucasians and the principal route of infection was through IVDU. The samples were all either Gt1 or Gt3 (Table 1).

### 3.4. E1E2 Binding of Chronic Cohort

The sera were screened for binding to H77 gt1a E1E2 lysate and in parallel to the negative control lysate. The same positive control for normalization between experiments was included on the ELISA plate. For this group, perhaps not surprisingly as they have an ongoing HCV infection, we found much stronger binding to E1E2. In addition, a higher proportion of the cohort (38/41 (92.7%)) had >10% binding to H77 gt1a E1E2 lysate (Figure 1b).

### 3.5. Neutralization of Chronic Cohort

**Figure 2:**
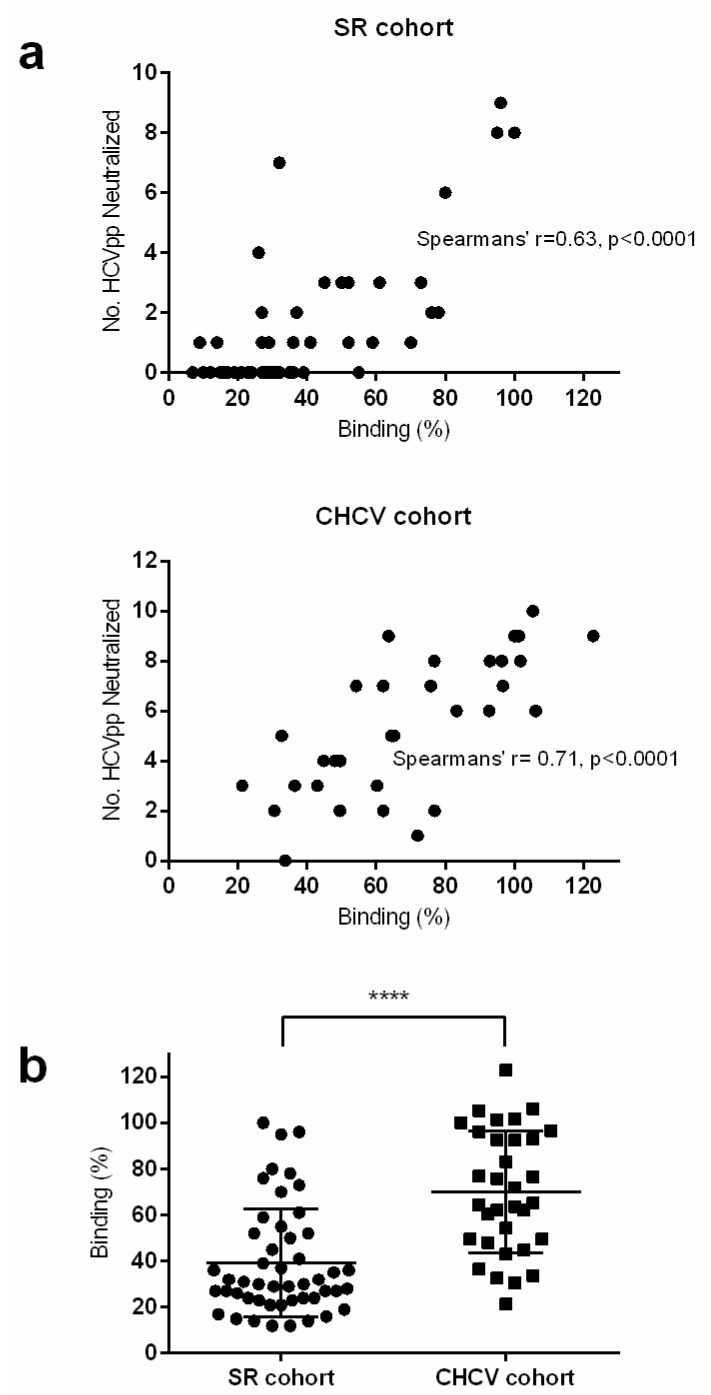
Correlation of binding and neutralization. (**a**) For each sample, the relative binding (%) to gt1a H77 E1E2 lysate was plotted against the number of HCVpp that were neutralized. SR cohort (top panel) and CHCV cohort (lower panel). Spearman’s rho correlation was plotted for both graphs. (**b**) The relative binding (%) to gt 1a H77 E1E2 lysate of samples within both cohorts were plotted. A parametric *t*-test was used to compare binding between both groups, (**** denotes that the p-value is less than 0.0001).

To enable comparison of the neutralization activity between the two cohorts, the IgG was purified from the strongest binding chronic sera (33) and neutralization of the Gt1 HCVpp panel was assessed under the same conditions. Similar to the observations for E1E2 binding, overall the chronic cohort was much more potent for neutralization. All chronic sera could neutralize at least one HCVpp isolate by at least 50% (Table 3) and more than half the cohort (18/33) was classified as broadly neutralizing. Interestingly, but perhaps not surprisingly, in both cohorts we found a significant correlation between the level of E1E2 binding and the neutralization activity of the sera (Figure 2a). In addition, there was a highly significant difference between the E1E2 binding of the cohort samples, this may explain why the chronic sera have stronger neutralization activity overall (Figure 2b).

### 3.6. Analysis of Neutralizing Epitopes

To address the question of whether the generated antibody response has enabled clearance of the virus because it is fundamentally distinct from the ‘failed’ antibody response during chronic infection we investigated the neutralizing epitopes targeted by sera from both cohorts. We analysed all the samples in the SR cohort that neutralized at least two HCVpp. For the CHCV cohort we randomly selected a pair of sera that neutralized two to nine HCVpp respectively. The epitopes targeted by many bNAbs have been reported in the literature. Most of these epitopes lie on the neutralizing face of the E2 molecule [31,32]. Three important regions each bound by several bNAbs are Domain E (E2 aa412-423), Domain D (E2 aa 420-428, 441-443, 616) and Domain B (E2 aa 431-439, 529-535). Within these regions it is notable that particular highly conserved residues are recognised by multiple bNAbs. We created a small group of E1E2 mutants, in a gt1a H77 background, each with a key epitope-binding residue mutated to alanine. Within Domain E we selected L413 and W420, for Domain D, L441 and F442 and finally for Domain B, W539 and G530. Cell lysates containing the mutant E1E2 proteins were produced and used in a GNA-ELISA. The binding of purified IgG to wildtype (wt) gt1a H77 E1E2 and the E1E2 carrying the mutant epitope was measured and expressed relative to the wt gt1a H77 E1E2 control (Table 4). The Domain E mutants did not strongly inhibit binding of any sera, however several sera, bound more strongly (>150%) to L413A and W420A. This suggests that there are no antibodies binding directly to these residues in the sera, at least at a level sufficient for detection in this assay. The increase in binding suggests that mutation of L413A or W420A has altered the conformation of E1E2 such that some epitopes are more available, most likely within the E2 hypervariable region 1 (HVR-1) which is immediately upstream to Domain E. In contrast, mutation within the Domain D region, particularly at residue L441 reduced binding of the majority of sera in both cohorts. This suggests that within each serum, Domain D-binding antibodies are present. More interesting are the observations for Domain B, in both groups, mutation at either W529 or G530 reduced E1E2 binding indicating the presence of antibodies that bind this region. However, the G530A mutation has a significantly greater effect on the sera in the spontaneous resolver cohort than sera from the chronic cohort. This suggests that the spontaneous resolver cohort has more antibodies binding to Domain B.

In order to investigate further the epitopes bound by serum IgGs from both cohorts we performed cross-competition ELISA analysis with a group of well-characterized monoclonal antibodies (mAbs) that target different epitope regions; Domain A (CBH-4B), Domain B (HC-1, HC-11, 1.7), Domain C (CBH-7) and Domain E (HC33.1). Importantly, we assessed the cross-competition activity of each mAb against itself and the other mAbs (Table 5). As expected, in all cases the strongest competition was observed between each mAb and itself. We observed similar trends in both cohorts; none of the sera tested could compete with antibody to Domain E (HC33.1). In both cohorts there were one or two samples that could compete with the remaining antibodies targeting different regions, the non-neutralizing Domain A (CBH-4B) and Domain C (CBH-7). The majority of cross-competition between mAbs and the purified IgG from the sera was observed against the Domain B antibodies (HC-1, HC-11, 1.7). Similarly, 8/15 (53%) of SR cohort samples and 10/17 (58%) of the CHCV cohort samples could significantly inhibit binding of at least one of the Domain B antibodies (HC-1, HC-11, 1.7). A slightly higher proportion of the SR cohort (7/15 (47%)) compared to (7/17 (41%)) of the CHCV cohort samples could not cross-compete significantly with any of the mAbs tested even though they could effectively neutralize at least two different HCVpp suggesting that they contained nAbs that bound other epitopes. The most interesting of these was C1001 which could still neutralize seven different HCVpp even though it did not cross-compete with any of the mAbs tested.

## 4. Discussion

Our study of the neutralizing antibody responses of a large retrospective cohort of individuals that have spontaneously cleared HCV infection found that most of the SR Cohort samples did not contain detectable levels of E1E2 glycoprotein antibodies. This could be due to multiple reasons; firstly, there are examples of spontaneous clearance samples where nAbs could not be detected [9], secondly, it has been shown that while HCV antibodies can be detected within weeks of infection, antibodies to E1E2 could not be detected until much later timepoints [9,16]. In addition, studies have also shown that the antibody response wanes over time post-clearance, presumably due to lack of the presence of viral antigen [16,33] therefore even if nAbs were present initially, they may no longer be detectable as in many cases samples were collected up to several years post-clearance. Comparison of the level of detectable E1E2 glycoprotein antibodies in the sera was lower in the SR cohort than that detected in samples taken from individuals with a persistent chronic infection. Again, this is consistent with previous reports whereby the level of antibody detected in spontaneous clearers was lower than in chronic infection [9,16,34].

Similarly, when we investigated the neutralizing response against a panel of diverse Gt1 HCVpp we found that the SR cohort had lower levels of neutralization overall, compared to the CHCV cohort. This is in concordance with a previous study [9]. This could simply be a consequence of the lower levels of E1E2 binding antibodies detected in the SR cohort, indeed, we observed a strong correlation between the level of E1E2 binding and the level of neutralization. Alternatively, the lower levels of neutralization may be indicative of the breadth of neutralizing response present in the samples. We are unable to assess the level of autologous neutralization in the SC cohort samples as we do not have virus from the individuals, indeed in many cases we do not even know what genotype they were infected with. Therefore, we are reliant on assessing how efficiently the antibodies present can neutralize a heterologous, diverse panel of HCVpp. It is possible that for those individuals that cleared early during infection, they mounted effective neutralizing responses against autologous virus but as this was cleared the antibody response did not mature and diversify over time to become efficient at neutralizing different viruses. However, this is perhaps less likely as our cohort is composed of individuals that have cleared during both acute and chronic infection.

Further investigation to characterize the type of neutralizing response for both cohorts using mutagenesis and mAb cross-competition approaches showed broadly similar results for both cohorts. Interestingly, in the mutagenesis study the SR cohort samples were more sensitive to mutation of G530A in Domain B than the CHCV cohort samples. While in the cross-competition analysis, samples within both cohorts could effectively compete with the Domain B antibodies. The mutagenesis analysis indicates that a higher proportion of E1E2 antibodies present in the SR cohort bind to this region than in the CHCV cohort. Although the cross-competition analysis suggests that even though antibodies recognizing Domain B appear to be less prevalent in the CHCV samples, they are more efficient at cross-competing with the Domain B mAbs. This implies that the CHCV Domain B antibodies, though fewer, may have higher affinity for this epitope. Investigation of Domain E by mutagenesis or cross-competition analysis did not indicate the presence of antibodies binding to this region in either cohort. This is perhaps not surprising as it is well-documented that Domain E antibodies are rare [35]. Although interestingly, mutation of Domain E did increase E1E2 binding in several samples in both cohorts, this is most likely caused by these mutations making other E1E2 epitopes more accessible. The domain E region is notably a very flexible region of the E2 protein [36,37,38,39], thus mutagenesis may alter the conformation of the protein [40]. Mutagenesis analysis also indicated that most of the samples in both cohorts developed antibodies that could bind to domain D of E2. Domain B and Domain D antibodies both bind to the neutralizing face of the E2 protein and inhibit the critical interaction of E2 with the CD81 receptor, indeed the epitopes for Domain B and D overlap [21,41]. Only one sample in each cohort could compete with CBH-7 which binds to the Domain C region of E2, and this is also on the neutralizing face but does not overlap with Domains B and D [29]. Similarly, only three samples could compete with the non-neutralizing antibody CBH-4B which binds to Domain A.

A limitation of our analysis of the antibody response is that using the mutagenesis approach and the cross-competition analysis we were confined to those nAbs that have well-documented epitopes and were suited to the cross-competition assay. Consequently, with these methods we cannot detect nAbs that bind other regions of the E1E2proteins. These may be epitopes that have already been described for other HCV nAbs such as the AR4A mAb which recognises the E1E2 heterodimer [42], the recently described E1 nAbs [43], or novel nAbs which are yet to be described. We do, however, observe in both cohorts, samples that could neutralize HCVpp but could not cross-compete with any of the mAbs tested indicating the presence of different nAbs in these samples. While the mutagenesis analysis indicates Domain D antibodies in these samples, it is likely that other nAbs are also present.

Our study of nAb responses of individuals that have spontaneously cleared infection or are chronically infected with HCV has identified similar profiles of nAb responses in both cohorts. This suggests that the neutralizing responses that succeed in clearing virus infection are similar to those that ‘fail’ and lead to chronic infection. The fact that samples from both cohorts had varied nAb profiles also indicates that with the methodological approaches described in this study, there is no singular immune response that is required for clearance, suggesting that multiple factors likely contribute to clearance vs. chronicity. The timing of the antibody response has been suggested to be important both in clearance of acute infection and chronic infection [11,16,18]. Furthermore, development of nAbs stimulates virus evolution to escape the immune pressure, which in some cases has been shown to result in reduced viral ‘fitness’ [30,44,45,46]. Thus, it is probable that a complex interplay between host and virus dictates the outcome of infection.

Our findings agree with other studies that have investigated this question by other methods. Keck et al. (2019) analysed in detail broadly neutralizing antibodies present in an individual that had cleared multiple HCV infections and concluded that the specificity of the antibody response was similar to that of a chronic infection [47]. Equally, Bailey et al. (2017) isolated nAbs from a spontaneous clearer and showed that even though the antibodies had fewer somatic mutations than those isolated from chronic infection, the epitopes of these nAbs mapped to Domains B and D [48]. A further study by Eliyahu et al. (2018) and colleagues analysing the antibody repertoire of spontaneous clearers concluded that nAbs were different compared to those found in chronic infection in that they had fewer somatic mutations [49]. This agrees with the report by Bailey et al. (2017), although critically, while Eilyahu et al. (2018) confirmed that antibodies from spontaneous clearers were broadly neutralizing they did not identify the epitopes that were targeted [48,49]. These data together with our findings bode well for the potential development of an effective HCV vaccine in that it seems likely that a vaccine does not need to elicit a highly specific nAb or nAb profile.

## Figures and Tables

**Table 1:** Cohort demographics.

Demographics		SR Cohort (233)	CHCV Cohort (41)
Age (yrs)	Median (Range)	45 (20–80)	46 (34–68)
Gender (M/F)	No. male (%)	156 (66.9%)	28 (68.3%)
Ethnicity	No. Caucasian (%)	212 (91%)	37 (90.2%)
Source of infection	No. IVDU (%)	159 (68.2%)	26 (63.4%)
Estimated duration (yrs)	Median (Range)	2.15 (0–20.2 ^a^)	29 (2–58 ^b^)
Genotype	No. Gt1, No. Gt3	Gt1 (12), Gt3 (13) ^c^	Gt1 (23), Gt3 (18)

^a^ no data for 122 subjects, ^b^ no data for 10 subjects, ^c^ no data for 199 subjects.

**Table 2:** Neutralization activity of SR cohort. The relative neutralization activity (%) of the Gt1 panel is shown. The data is the mean value from triplicate independent experiments.

Sera ID	Relative Neutralization (%) of HCVpp in Panel Gt1 ^a^											No.HCVpp Neutralized by >50%
	H77	UKN1B5.23	UKN1A14.38	UKN1A14.43	UKN1B14.818	UKN1A20.8	GC12.02	GC13.01	GC34.11	GC.37.04	ET10	
S935	**68^11^**	35^6^	**65^0^**	**65^2^**	**79^4^**	31^16^	**61^2^**	**69^2^**	**66^14^**	**55^3^**	**68^3^**	9
S37	**67^5^**	39^10^	**61^3^**	**52^1^**	**75^4^**	38^6^	**57^8^**	39^9^	**58^2^**	**63^0^**	**65^3^**	8
S922	**72^1^**	33^10^	**68^1^**	**51^4^**	**72^5^**	30^8^	**56^9^**	43^5^	**63^1^**	**54^4^**	**66^11^**	8
S25	**61^2^**	31^14^	42^4^	34^1^	**83^4^**	33^11^	**61^13^**	**61^5^**	**50^1^**	**50^3^**	**54^0^**	7
S887	**62^3^**	25^4^	47^3^	**52^1^**	**59^3^**	16^1^	43^3^	41^8^	**56^3^**	**51^0^**	**59^2^**	6
S107	**66^5^**	39^9^	38^8^	32^10^	**61^3^**	21^6^	49^6^	25^11^	**54^7^**	**58^4^**	48^4^	4
S9	**53^4^**	25^8^	41^4^	43^2^	44^4^	30^5^	**56^8^**	41^8^	**53^3^**	41^3^	39^3^	3
S12	**53^8^**	32^1^	34^3^	36^10^	**52^9^**	27^10^	43^2^	13^18^	**52^6^**	44^4^	37^2^	3
S78	**53^10^**	44^9^	37^4^	19^3^	**58^6^**	31^9^	39^5^	−6^21^	39^4^	41^5^	53^7^	3
S197	**61^4^**	18^5^	32^7^	32^17^	46^4^	32^12^	**52^7^**	21^15^	**54^4^**	40^1^	41^4^	3
S934	37^7^	34^6^	36^4^	34^1^	**62^3^**	30^6^	47^2^	29^2^	**57^2^**	53^5^	47^1^	3
S10	45^13^	−3^4^	34^3^	26^6^	**59^4^**	−14^5^	35^8^	30^9^	**52^0^**	38^6^	31^3^	2
S15	48^9^	25^6^	42^4^	47^4^	**58^2^**	25^3^	32^7^	22^1^	**59^7^**	37^7^	34^4^	2
S58	**60^4^**	40^9^	34^5^	33^2^	**55^6^**	43^3^	48^5^	29^10^	40^8^	48^1^	40^1^	2
S893	43^1^	25^9^	35^6^	33^2^	**52^5^**	12^10^	**51^1^**	28^10^	37^11^	45^1^	40^7^	2
S135	39^2^	27^9^	14^3^	26^3^	**54^2^**	25^10^	40^6^	12^11^	32^1^	41^3^	35^2^	1
S151	41^5^	18^0^	22^6^	13^1^	**59^9^**	23^17^	35^8^	15^6^	38^1^	36^11^	31^3^	1
S888	23^3^	13^8^	4^2^	16^4^	**58^9^**	24^23^	33^1^	−13^26^	32^6^	32^8^	37^1^	1
S890	31^7^	20^6^	27^1^	26^10^	**54^6^**	4^23^	31^17^	35^4^	32^0^	42^6^	31^6^	1
S924	**59^1^**	14^7^	22^8^	36^6^	44^6^	31^16^	35^1^	31^15^	35^2^	39^7^	29^5^	1
S927	36^9^	4^22^	28^2^	22^6^	**51^5^**	−1^7^	42^5^	6^15^	12^26^	41^3^	29^10^	1
S930	36^1^	21^19^	24^7^	20^15^	**50^2^**	6^1^	34^6^	26^9^	38^9^	33^2^	29^9^	1
S939	33^7^	21^12^	32^6^	17^17^	**58^7^**	10^15^	32^11^	20^8^	31^7^	44^6^	42^4^	1
S948	41^1^	35^0^	33^4^	28^11^	**60^6^**	39^1^	42^3^	26^2^	39^2^	42^0^	36^3^	1
>50%	12	0	3	4	21	0	7	2	12	6	5	
<20%	0	6	2	4	0	7	0	6	1	0	0	

^a^ Values in bold show the neutralization of HCVpp >50% and values that are underlined show neutralization of HCVpp <20%. The superscript number indicates the standard error of the mean (SEM).

**Table 3:** Neutralization of CHCV cohort. The relative neutralization activity (%) of the Gt1 panel. The mean value from triplicate independent experiments is shown.

Sera ID	Relative Neutralization (%) of HCVpp in Panel Gt1 ^a^											No.HCVpp Neutralized by >50%
	H77	UKN1B5.23	UKN1A14.38	UKN1A14.43	UKN1B14.818	UKN1A20.8	GC12.02	GC13.01	GC34.11	GC.37.04	ET10	
C1003 ^b^	**88**	**73**	**54**	**64**	**89**	25	**57**	**55**	**53**	**84**	**90**	10
C1013 ^b^	**86**	17	**67**	**72**	**81**	37	**61**	**58**	**89**	**81**	**88**	9
C1023 ^b^	**84**	35	**56**	**68**	**72**	17	**74**	**65**	**76**	**71**	**77**	9
C1032 ^b^	**86**	44	**81**	**82**	**90**	28	**57**	**70**	**89**	**74**	**74**	9
C1035 ^b^	**78**	43	**61**	**62**	**76**	45	**67**	**65**	**71**	**67**	**83**	9
C1012 ^b^	**80**	34	**76**	**58**	**87**	24	**54**	**50**	**67**	**84**	**79**	8
C1016	**87^5^**	**55^5^**	**66^5^**	49^4^	**94^2^**	44^23^	**50^8^**	40^5^	**63^7^**	**75^8^**	**89^7^**	8
C1031 ^b^	**77**	37	**56**	**56**	**70**	28	46	**64**	**72**	**72**	**68**	8
C1042	**78^7^**	^2^	**57^8^**	**59^9^**	**77^2^**	11^22^	**58^6^**	48^9^	**61^10^**	**76^7^**	**77^5^**	8
C1001 ^b^	**69**	14	**72**	**61**	**81**	11	29	45	**52**	**53**	**73**	7
C1022 ^b^	**77**	39	49	46	**65**	24	**55**	**53**	**55**	**66**	**76**	7
C1036 ^b^	**79**	41	42	**50**	**57**	28	**51**	44	**55**	**69**	**70**	7
C1046	**69^9^**	27^2^	**53^7^**	**57^4^**	**74^1^**	22^13^	**50^10^**	49^1^	**53^10^**	**59^8^**	**43^4^**	7
C1010 ^b^	**78**	31	46	**56**	**87**	18	44	41	**55**	**77**	**70**	6
C1021	**79^4^**	**60^5^**	36^9^	**50^4^**	**69^3^**	15^11^	**50^13^**	37^9^	**56^8^**	**60^5^**	**52^9^**	6
C1037 ^b^	**94**	28	**54**	34	77	16	45	41	**79**	**62**	**75**	6
C1050	**77^8^**	39^2^	26^13^	25^17^	**66^5^**	6^16^	**52^8^**	40^4^	**56^8^**	**51^4^**	**53^6^**	6
C1029	**78^8^**	34^5^	29^5^	26^17^	**66^6^**	18^9^	37^10^	18^18^	**56^8^**	**57^4^**	**52^6^**	5
C1038	**66^7^**	35^1^	37^2^	**50^2^**	**62^7^**	14^5^	30^12^	28^7^	**54^7^**	**54^2^**	**60^8^**	5
C1061	**52^7^**	28^4^	18^3^	16^6^	**63^7^**	15^14^	38^4^	37^14^	**61^10^**	**53^5^**	**54^4^**	5
C1015	**51^10^**	28^8^	21^14^	41^6^	**62^4^**	17^12^	28^9^	21^11^	45^1^	**51^2^**	**62^10^**	4
C1055	**58^5^**	27^13^	**58^2^**	**51^11^**	**58^4^**	12^18^	10^9^	15^12^	33^3^	45^4^	31^11^	4
C1056	**56^11^**	21^3^	21^8^	22^8^	**61^6^**	6^20^	41^17^	32^3^	42^5^	**54^11^**	52^2^	4
C1018	**53^1^**	32^1^	2^012^	27^11^	**58^6^**	2^011^	26^12^	4^010^	**51^6^**	37^6^	35^11^	3
C1030 ^b^	**58**	45	27	20	**63**	16	23	20	21	48	**60**	3
C1045 ^b^	**70**	12	29	28	**70**	0	33	30	**53**	48	49	3
C1060 ^b^	**51**	40	24	33	**58**	15	36	30	48	**56**	49	3
C1034 ^b^	47	24	24	24	**50**	18	32	4	22	**53**	42	2
C1049	**50^3^**	33^2^	14^16^	24^17^	**55^2^**	14^13^	45^13^	8^9^	44^5^	49^7^	31^7^	2
C1054	**64^6^**	32^2^	14^6^	12^27^	**52^7^**	2^16^	44^13^	28^7^	43^2^	43^5^	40^7^	2
C1062	**57^5^**	24^5^	21^7^	20^11^	39^6^	16^10^	37^5^	42^12^	**50^3^**	42^4^	37^6^	2
C1040	30^11^	17^7^	−5^8^	5^21^	**52^6^**	−11^25^	21^7^	13^12^	−1^23^	47^12^	29^12^	1
>50%	30	3	13	15	31	0	13	8	23	24	22	
<20%	0	4	4	3	0	21	1	5	1	0	0	

^a^ Values in bold show the neutralization of HCVpp >50% and values that are underlined show neutralization of HCVpp <20%. The superscript number indicates the standard error of the mean (SEM). ^b^ Neutralization data for many Gt1 samples in the CHCV cohort has been published previously [24].

**Table 4:** E1E2 mutagenesis binding analysis.

	Sample	Domain E		Domain D		Domain B		No. HCVpp Neutralized
		L413A	W420A	L441A	F442A	W529A	G530A	
SPONTANEOUS RESOLVER	SR935	103^2^	98^1^	64**^1^**	90^4^	95^4^	80**^0^**	9
	SR37	98^1^	98^2^	64**^2^**	96^3^	103^2^	89^4^	8
	SR922	99^3^	99^2^	76**^0^**	111**^2^**	110^2^	89^5^	8
	SR25	113^6^	103^3^	47**^3^**	80^9^	76^10^	77**^1^**	7
	SR887	108^8^	100^4^	62**^13^**	84^20^	81^16^	69**^0^**	6
	SR107	110^4^	109^4^	73**^8^**	108^4^	110^5^	28**^5^**	4
	SR9	115**^4^**	101^5^	60**^12^**	94^16^	86^8^	58**^2^**	3
	SR12	112^7^	101^4^	55**^9^**	71^13^	78^10^	73**^2^**	3
	SR78	105^4^	137^5^	82^8^	116**^5^**	126**^4^**	60**^8^**	3
	SR197	113**^4^**	121**^4^**	67**^4^**	101^8^	97^11^	41^4^	3
	SR934	154**^12^**	121^8^	49^1^	69^5^	110^8^	48^1^	3
	SR10	82**^8^**	80^10^	49^7^	71^11^	71^9^	66^2^	2
	SR15	119^8^	135**^12^**	67**^10^**	119^16^	133^19^	80^6^	2
	SR58	124**^6^**	108**^1^**	43**^4^**	77^8^	87^7^	61^1^	2
	SR893	138**^5^**	123**^3^**	10^1^	60^3^	76^3^	64^6^	2
CHRONIC	C1003	108^7^	116^7^	66^3^	110^5^	115^4^	93^3^	10
	C1013	106^10^	109^10^	51^1^	112^8^	114^7^	87^5^	9
	C1035	100^11^	98^9^	50^6^	111^16^	117^20^	99^13^	9
	C1012	96^8^	99^10^	57^8^	91^14^	82^11^	85^4^	8
	C1042	108^9^	118^8^	50^4^	104^6^	106^9^	82^4^	8
	C1001	112^12^	116^6^	44^3^	105^4^	110^2^	74^3^	7
	C1022	83^11^	105^11^	80^10^	104^9^	110^8^	91^19^	7
	C1010	101^12^	106^12^	67^6^	104^5^	112^0^	111^19^	6
	C1050	163^7^	189^12^	101^4^	162^11^	129^7^	104^8^	6
	C1038	108^3^	117^9^	59^2^	112^6^	115^7^	81^3^	5
	C1029	154^5^	173^8^	78^5^	158^10^	140^5^	128^5^	5
	C1055	220^17^	244^15^	57^7^	88^6^	94^3^	72^5^	4
	C1056	180^6^	213^6^	51^3^	75^2^	80^2^	48^3^	4
	C1018	86^14^	101^12^	73^1^	115^15^	116^4^	89^4^	3
	C1045	102^7^	119^8^	48^1^	121^7^	134^1^	97^9^	3
	C1049	185^17^	135^12^	11^5^	33^2^	31^3^	24^4^	2
	C1054	140^22^	148^10^	15^5^	57^5^	54^9^	43^4^	2
	ALP98	102^4^	112^7^	130^9^	111^8^	134^11^	123^7^	Control

The relative binding (%) of purified IgG to a group of E1E2 lysates containing a single alanine mutation of key antibody binding residues. The mean value of three independent experiments is shown. The superscript number indicates the standard error of the mean (SEM). The values are shaded to reflect the level of binding compared to the wt control (brown = 0–30%, orange = 31–50%, light orange = 51–80%, white = 81–120%, light blue = 121–150%, blue = >150%).

**Table 5:** Cross-competition analysis with neutralizing mAbs.

		Biotinylated Antibody						No. HCVpp Neutralized
		CBH-7	HC-1	HC-11	1.7	HC33.1	CBH-4B	
**Control**	CBH-7	97.8^1^	68.0^2^	82.5^1^	86.5^2^	−5.6^8^	82.2^8^	
	HC-1	12.1^4^	96.2^1^	93.0^2^	97.7^0^	1.1^6^	34.7^16^	
	HC-11	7.5^5^	39.4^5^	92.9^1^	82.3^1^	12.3^15^	12.0^7^	
	1.7	10.1^6^	54.9^8^	87.9^4^	88.6^3^	23.6^23^	11.5^6^	
	HC33.1	23.7^2^	43.3^8^	54.3^11^	57.8^8^	86.2^5^	31.3^4^	
	CBH-4B	10.2^1^	9.4^7^	24.7^7^	22.3^6^	22.1^10^	95.1^1^	
**Spontaneous resolvers**	S935	30.5^9^	64.1^5^	82.7^5^	78.4^5^	1.9^5^	40.7^6^	9
	S922	64.6^4^	62.3^6^	72.7^9^	77.7^4^	−1.3^5^	39.9^7^	8
	S37	31.5^10^	64.3^6^	59.5^11^	68.6^9^	−7.5^1^	13.6^5^	8
	S25	5.0^4^	36.9^9^	56.7^15^	52.7^10^	−1.8^3^	11.3^4^	7
	S887	23.4^9^	48.4^10^	63.9^8^	68.1^9^	6.4^8^	57.0^6^	6
	S107	11.7^1^	13.7^2^	32.0^3^	32.4^4^	−22.2^4^	27.1^3^	4
	S12	5.0^1^	16.2^3^	35.3^5^	32.9^10^	−10.7^1^	−5.3^5^	3
	S934	5.8^3^	15.7^2^	30.5^5^	33.2^7^	−11.3^2^	8.9^4^	3
	S9	10.3^2^	23.0^2^	24.7^6^	26.1^5^	−5.1^2^	15.8^4^	3
	S78	38.1^4^	48.2^1^	74.1^2^	63.0^3^	−4.0^2^	63.3^4^	3
	S197	20.1^2^	48.5^2^	70.1^2^	61.2^1^	−9.4^1^	23.6^5^	3
	S58	0.3^3^	10.3^4^	4.9^3^	21.9^1^	-23.3^10^	13.4^4^	2
	S15	12.4^3^	18.0^2^	25.8^4^	27.9^2^	−6.3^6^	11.8^6^	2
	S10	28.8^5^	31.9^1^	50.6^3^	49.9^3^	−17.0^3^	32.6^3^	2
	S893	1.1^3^	13.9^4^	11.0^9^	21.0^5^	−8.4^7^	−3.4^4^	2
**Chronic**	C1003	19.7^2^	59.2^3^	79.0^8^	79.5^6^	−7.5^5^	1.6^5^	10
	C1013	2.0^3^	44.4^7^	64.3^8^	70.1^2^	0.8^6^	12.0^9^	9
	C1035	23.9^7^	47.9^4^	68.8^2^	58.5^7^	0.5^5^	−4.3^4^	9
	C1012	45.6^7^	55.3^5^	78.9^4^	80.2^2^	15.2^7^	54.9^4^	8
	C1042	9.6^4^	74.9^2^	94.5^2^	89.2^1^	6.1^1^	23.8^8^	8
	C1022	26.3^2^	21.8^4^	92.0^2^	87.3^0^	−3.6^1^	22.4^9^	7
	C1001	18.9^9^	20.9^4^	8.4^6^	24.9^7^	3.2^3^	0^8^	7
	C1010	54.6^1^	56.0^2^	89.3^1^	87.4^1^	−6.9^1^	34.3^14^	6
	C1050	17.1^7^	66.0^5^	67.4^6^	74.7^5^	−0.2^7^	32.4^7^	6
	C1038	12.7^2^	16.4^1^	14.0^6^	16.1^6^	6.4^8^	0.6^4^	5
	C1029	13.0^7^	65.9^6^	80.9^5^	76.9^6^	0^8^	19.1^3^	5
	C1055	7.7^4^	25.9^9^	32.2^6^	37.7^8^	5.5^4^	13.0^7^	4
	C1056	7.8^2^	57.1^3^	70.4^5^	70.0^4^	7.7^6^	14.2^1^	4
	C1018	−0.4^6^	19.3^3^	10.9^6^	32.4^6^	−10.5^8^	−1.7^5^	3
	C1045	8.0^3^	19.7^3^	12.5^4^	25.2^2^	0.6^3^	5.7^3^	3
	C1049	5.0^4^	30.7^5^	34.9^8^	49.2^3^	−4.2^6^	15.6^7^	2
	C1054	5.5^5^	39.9^6^	39.5^4^	51.1^2^	0^8^	16.3^5^	2

Competition of purified IgG against a panel of well-characterized control E2 mAbs (CBH-7, HC-1, HC-11, 1.7, HC33.1 and CBH-4B) was performed in triplicate. The mean percent competition, reduction in mAb binding in the presence of IgG is shown. The superscript number indicates the standard error of the mean (SEM). The values are shaded to reflect the level of reduced binding compared to the no antibody control. (brown = 90–100%, orange = 70–89%, light orange = 50–69%; white = 0–49%, light blue = −1– −19%, blue = >−20%).

## Data Availability

Not applicable.

## Supplementary Materials

The following supporting information can be downloaded at: https://www.mdpi.com/article/10.3390/v14071391/s1, Table S1: Clinical data of spontaneous resolvers with E1E2 binding activity.
