# Supplementary material for: The Neutralizing Antibody Responses of Individuals That Spontaneously Resolve Hepatitis C Virus Infection
Source: Viruses. 2022 Jun 25;14(7):1391. doi: 10.3390/v14071391 (PMC9318067; doi:10.3390/v14071391)
Supplement: Supplementary file 1 [file viruses-14-01391-s001.zip › viruses-1725829-supplementary.pdf]

Table S1. Clinical data of spontaneous resolvers with E1E2binding activity.

Sera ID	Gender	Birth Year	Ethnicity	Date 1st positive test	Earliest negative result	Date collection	HCV Gt	Est. yr of infection	Route infection
**S3**	M	1962	White	01.01.95	30.5.07	13.11.12	NR	1978	IDU
**S8**	F	1984	White	01.06.12	13.10.12	13.12.12	NR	1999	IDU
**S9**	M	1973	White	01.01.11	no PCR, ab+	19.12.12	NR	2001	IDU
**S10**	M	1980	White	01.01.08	12.6.12	03.01.13	1	NR	IDU
**S12**	M	1964	White	01.01.97	Ab+	14.01.13	3	1980	IDU
**S15**	M	1979	White	04.12.06	24.1.13	28.1.13	1	NR	IDU
**S24**	F	1981	White	30.03.11	Ab+	04.02.13	3	2011	IDU
**S25**	M	1972	White	02.12.98	13.10.12	11.02.13	NR	NR	IDU
**S32**	M	1962	White	30.11.12	Ab+	14.01.13	NR	1986	IDU
**S35**	M	1979	White	NR	23.05.11	11.03.13	NR	1998	IDU
**S37**	F	1954	White	04.09.09	Ab+	18.03.13	NR	NR	IDU
**S39**	M	1981	White	01.01.99	14.03.13	14.03.13	NR	1997	IDU
**S41**	M	1954	White	07.02.11	NR	26.03.13	2	2006	IDU
**S53**	M	1948	White	NR	25.11.11	24.05.13	NR	NR	IDU
**S58**	M	1979	Asian	01.01.11	08.09.13	08.09.13	NR	NR	Born abroad
**S75**	M	1964	White	14.11.04	25.08.00	09.01.13	NR	NR	Blood/ products
**S78**	M	1978	White	31.08.12	NR	03.12.13	NR	NR	IDU
**S93**	M	1989	White	23.08.13	09.12.13	27.01.14	NR	NR	IDU
**S95**	M	1955	White	27.11.92	24.08.09	24.01.14	NR	1970	IDU
**S102**	M	1966	White	01.01.93	NR	31.03.14	NR	NR	IDU
**S106**	F	1981	White	01.09.05	NR	11.06.14	1	2005	IDU
**S107**	M	1957	White	NR	NR	24.06.14	1	NR	IDU
**S112**	M	1984	White	NR	26.06.14	12.02.14	NR	2006	IDU
**S113**	M	1976	White	07.06.11	11.09.11	07.06.12	NR	NR	Tattoo
**S120**	M	1956	White	07.08.02	19.05.10	17.07.12	NR	1972	IDU
**S121**	F	1962	White	01.08.12	31.7.12	03.10.97	NR	1997	IDU
**S122**	M	1956	White	08.09.99	28.09.00	31.07.12	NR	1977	IDU
**S124**	M	1983	White	01.01.04	NR	19.06.14	NR	NR	IDU
**S125**	M	1977	White	NR	NR	20.06.14	NR	2012	IDU
**S135**	F	1947	Asian	20.06.11	NR	08.09.14	3	NR	Born abroad
**S151**	M	1966	White	01.01.06	12.06.13	26.06.13	other	2003	IDU
**S153**	M	1980	White	30.04.13	NR	04.07.13	NR	NR	IDU
**S156**	F	1983	White	07.06.11	31.01.12	18.07.13	NR	NR	IDU
**S197**	F	1947	Asian	20.06.11	NR	08.09.14	3	NR	Born abroad
**S887**	M	1973	White	30.08.11	NR	26.08.13	NR	NR	IDU
**S888**	M	1983	Other	01.04.13	NR	29.08.13	4	2013	IDU
**S889**	M	1986	White	NR	NR	29.08.13	3	NR	IDU
**S890**	M	1979	White	01.01.12	18.09.13	03.12.13	3	NR	Prison
**S893**	M	1960	White	01.08.01	11.12.08	10.10.13	NR	NR	IDU
**S900**	M	1948	White	01.01.97	NR	22.11.13	NR	1965	IDU
**S906**	F	1976	Asian	09.11.08	27.11.13	27.11.13	NR	NR	Born abroad
**S922**	F	1972	White	16.07.10	NR	17.10.14	NR	NR	IDU
**S924**	M	1971	White	01.01.13	NR	02.09.14	1	1991	Other
**S927**	M	1974	White	02.02.07	NR	08.05.14	NR	1999	IDU
**S930**	M	1961	White	21.01.13	28.02.13	13.06.14	3	2010	IDU
**S934**	M	1969	Other	28.07.03	NR	04.03.14	NR	NR	Born abroad
**S935**	M	1969	White	27.09.07	NR	04.03.14	NR	NR	IDU
**S939**	F	1991	White	20.03.14	NR	12.08.14	NR	2008	Blood/ products

**S948**	M	1959	Mixed	14.03.07	NR	25.07.14	NR	2003	IDU
